# Fatigue-Limit Assessment via Infrared Thermography for a High-Strength Steel

**DOI:** 10.3390/ma18020279

**Published:** 2025-01-10

**Authors:** Yingxin Zhao, Zhaodong Lin, Yu Xia, Liming Chen, Guoqing Gu, Like Pan

**Affiliations:** 1 Standards & Metrology Research Institute, China Academy of Railway Sciences Corporation Limited, Beijing 100010, China; yxzhao15801042226@163.com (Y.Z.); 13810305638@163.com (L.C.); 2 College of Civil Engineering, Nanjing Tech University, Nanjing 211816, China; linzd727@163.com (Z.L.); xiayu@njtech.edu.cn (Y.X.); 3 College of Civil Engineering, Yancheng Institute of Technology, Yancheng 224051, China; gqgu@ycit.edu.cn

**Keywords:** high-cycle fatigue, intrinsic dissipation, infrared thermography, fatigue limit, temperature variation

## Abstract

Infrared thermography techniques have proven to be very effective for assessing the fatigue limits of metallic materials with obvious temperature variations. But for some materials, it has been shown that the temperature variation is very limited, and the accuracy of infrared thermographic techniques is not verified. In this study, the fatigue properties of a high-strength steel (SAE52100) were evaluated with traditional fatigue-loading techniques and infrared thermographic methods. The traditional fatigue experiments were loaded at a frequency of 80 Hz with a stress ratio of *R* = −1, and the fatigue limit at the fatigue lifetime of N = 10^7^ cycles was about 800 MPa. Besides, three additional specimens were loaded with step-by-step increasing stress-loading amplitude, where the maximum temperature increments and temperature distribution were recorded via infrared thermographic techniques. The infrared detections revealed that the maximum value of the temperature increase was only about 1 °C. The fatigue limit was first evaluated based on the maximum temperature variation, then the prediction was refined based on fatigue intrinsic dissipation. The fatigue limits predicted with maximum temperature variation were shown to be 841 MPa, 772 MPa, and 787 MPa, respectively, while the fatigue limits predicted based on fatigue intrinsic dissipation were 793 MPa, 791 MPa, and 789 MPa. Finally, an FEM simulation of temperature variation during fatigue loading was implemented to verify the experimental results. This study provides a solid foundation for the applications of infrared thermography techniques for materials with lower energy dissipations.

## 1. Introduction

Most mechanical components are subjected to cyclic loading during service time, which makes fatigue rather than static much more important in mechanical design. For many key components of engineering structures that need to be utilized for many years, fatigue limits of materials are adopted as design standards. Thus, the evaluation of fatigue limits for each material is vital in many industrial applications [1,2,3,4,5]. Fatigue limit refers to the maximum stress amplitude that occurs after infinite fatigue-loading cycles without failure, also known as the endurance limit. For materials with a horizontal asymptote S–N curve, such as structural steels, if the specimens have undergone 10^7^ stress cycles without fracture, they are considered to be able to undergo infinite stress cycles without failure, and the maximum stress is defined as the fatigue limit. Generally speaking, fatigue limits are usually defined as the stress level without fracture at fatigue lifetimes of N = 10^7^ cycles. Take a loading frequency of 50 Hz as an example, a specimen with fatigue lifetimes of N = 10^7^ cycles will consume about 56 h. Besides, at least 20 specimens are required to obtain an S–N curve. Therefore, fatigue experiments are time-consuming and expensive processes. To obtain fatigue properties quickly, many studies have been carried out in recent decades.

Accelerating fatigue experiments with much higher loading frequencies are first adopted for quick assessment of fatigue properties. Ultrasonic fatigue testing with a loading frequency of 20 kHz has been proposed since the 1980s [6] and has been widely utilized in the last decades [7,8,9]. Ultrasonic fatigue testing brought great convenient for fatigue property evaluations, while many controversies have been provoked due to the huge differences in loading frequencies and the following fatigue-damage accumulation mechanisms. Mayer et al. [10], Jeddi et al. [11], and Hong et al. [12] gave some literature reviews on the effects of loading frequency, which suggested that the loading frequency had no obvious effects on FCC and HCP metallic materials. But for bcc materials, ultrasonic fatigue loading will lead to obviously higher fatigue strength [13,14,15,16] as long as the temperature rise can be effectively suppressed.

During numerous fatigue tests, it is found that the temperatures of specimens rise obviously, and this phenomenon is named self-heating [17,18,19,20]. The fatigue self-heating effect is correlated with the energy dissipation during fatigue loading, where the dissipated energy is converted into heat and makes the temperature of specimens rise. The actual heating of the specimen is caused by the mechanical application of energy during the fatigue testing. As detected by infrared thermographic facilities, the temperature rise of a specimen during fatigue loading has three stages: initial temperature rise, temperature stabilization, and abrupt temperature rise leading to final failure. Moreover, the temperature increments for the temperature-stabilization stage depend on the loading-stress amplitude. It is assumed that temperature variation is correlated with the damage accumulation mechanism of fatigue. Thus, the temperature rise is utilized to assess the fatigue properties of materials.

In the beginning, temperature variations of specimens under different stress amplitudes during fatigue loading were utilized to predict fatigue limits. Rosa and Risitano et al. [20,21] collected the data of stable temperature increments under different stress amplitudes above the fatigue limit and fitted the data linearly, while the intersection of the fitted line with the stress horizontal axis (stress amplitude) was taken as the fatigue limit of the material. This method was named the “Risitano method”, in which step-by-step loading-stress amplitudes were conducted, and the increase in the maximum temperature value was recorded to obtain the relationship between stress level and stable temperature rise. Risitano et al. [21] verified that the accuracy of this method in evaluating fatigue limits can be controlled within 10% based on many experiments with different materials. Luong [22] found that there was still a small temperature rise when the stress amplitude was smaller than the fatigue limit. Therefore, linear fitting was performed on the experimental data below and above the fatigue limit, and the intersection of the two fitting lines was taken as the material fatigue limit. To distinguish from the method proposed by Risitano, the method was named the “Luong method” or “two-line method”. Compared to the traditional method for evaluating fatigue limits, this method based on temperature rise can greatly save experimental costs and durations. The two methods were verified and developed by many researchers [23,24,25,26] and proven to be very useful in fatigue-limit assessment. Moreover, the temperature variations are also adopted to assess the propagation of cracks [27,28,29,30,31].

However, the above studies are mostly based on phenomenological analysis of temperature variations, and the corresponding physical basis is not very clear. They are also greatly affected by many factors, such as the experimental environment, especially in open experimental environments, where the data are difficult to unify [32,33]. To overcome the shortcomings and deficiencies of utilizing temperature signals directly as fatigue-damage assessment indicators, many studies were carried out to convert temperature variations into energy dissipations, which were adopted to assess the fatigue performance of materials. Energy dissipation leading to temperature increase can be separated into several parts: energy dissipation due to the thermoelastic effect, energy dissipation due to the interactions between microstructures and temperature variation, and energy dissipation due to intrinsic dissipation [34,35,36]. It is acknowledged that the first two parts can be neglected in fatigue loading, while the last one, fatigue intrinsic dissipation, suggests the damage accumulation progresses at a microscopic scale. Fatigue intrinsic dissipation generally reveals the irreversible degradation of materials such as microplasticity caused by dislocation multiplication or plastic slip of crystal lattices [37,38,39]. Thus, the parameter of fatigue intrinsic dissipation rather than temperature increments is more reasonable for assessing fatigue-damage evolution. The accurate calculation of intrinsic dissipation is vital in fatigue-damage evolution assessment. To obtain accurate calculations of intrinsic dissipation, a strict thermal constitutive equation reflecting the process of fatigue loading should be established, and many models were developed by scholars. The developed methods can be divided into three types, one-dimensional models, zero-dimensional models, and rate-based models, and the differences mainly lie in the simplification of heat-diffusion equations [40,41,42]. Because of the different adopted simplifications, there were some deviations among different models for fatigue-limit predictions, but the predictions were proven to be closer to the traditional fatigue-testing results [43,44,45].

The reported works all indicated that the temperature variations of metallic materials are obvious during the fatigue-loading process, and the prediction results are very accurate. But for some materials, it is shown that the temperature increase is very limited, and the accuracy of infrared thermographic techniques is not verified. In this study, the fatigue properties of a high-strength steel (SAE52100) were evaluated with traditional fatigue-loading techniques and infrared thermographic methods. The typical fatigue experiments were conducted at a frequency of 80Hz, and the infrared detections revealed that the maximum temperature variation was only about 1 K. The fatigue limit was first evaluated based on variations in the maximum temperature value, then the prediction was refined based on fatigue intrinsic dissipation. Both the results were compared with the results of traditional fatigue experiments. Finally, an FEM simulation of temperature variation during fatigue loading was implemented to verify experimental results. This study would provide a solid foundation for the applications of infrared thermography techniques for materials with lower energy dissipations.

## 2. Materials and Methods

### 2.1. Materials and Specimens

**Figure 1:**
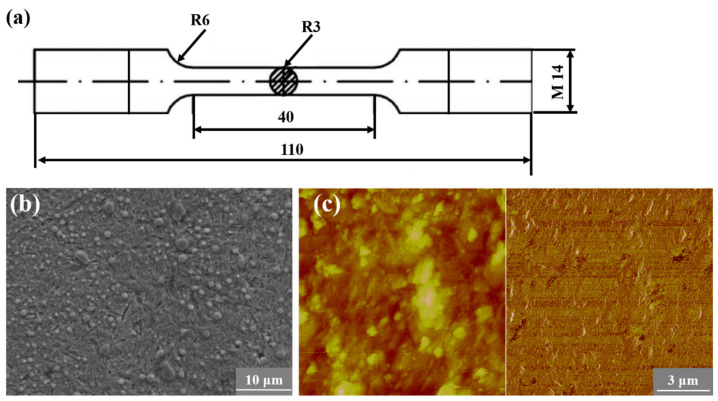
Specimens adopted in fatigue experiments and the corresponding microstructures. (**a**) Geometrical dimensions (dimensions: mm), (**b**) Scanning electron microscopy (SEM) micrographs, (**c**) atomic force microscopy (AFM) micrographs.

The metallic alloy used in this work is a high-strength-bearing steel, SAE52100, and the chemical composition is presented in Table 1. Cylindrical specimens were adopted for fatigue testing, as shown in Figure 1a, and were prepared with the Chinese standard “GB_T 228.1-2010” [46]. After rough machining, the raw specimens were first heated at 840 °C in a vacuum for 150 min, then oil-quenched and tempered in a vacuum at 300 °C for 120 min with furnace-cooling, and finally, they were fabricated to the final dimensions. The basic mechanical properties of the tested materials are shown in Table 2. The microstructures of the final specimens are shown in Figure 1b,c. It is seen that although the tempering temperature is relatively higher, some acicular martensite still exists. Besides, there is also clear evidence of small spheroidal carbides, which are evident in both the SEM photographs and the AFM photographs. The microstructure was observed by a field emission scanning electron microscope (SEM, MIRA 3 XMU, TESCAN, Brno, Czech Republic). The detailed surface topography of the microstructure was also investigated by an atomic force microscope (AFM, Bruker Dimension^®^ Icon™, Bruker, Karlsruhe, Germany). During AFM observations, the lens was adjusted to the specified zones with low magnification and then zoomed in to the target zones.

### 2.2. Experimental Methods

**Figure 2:**
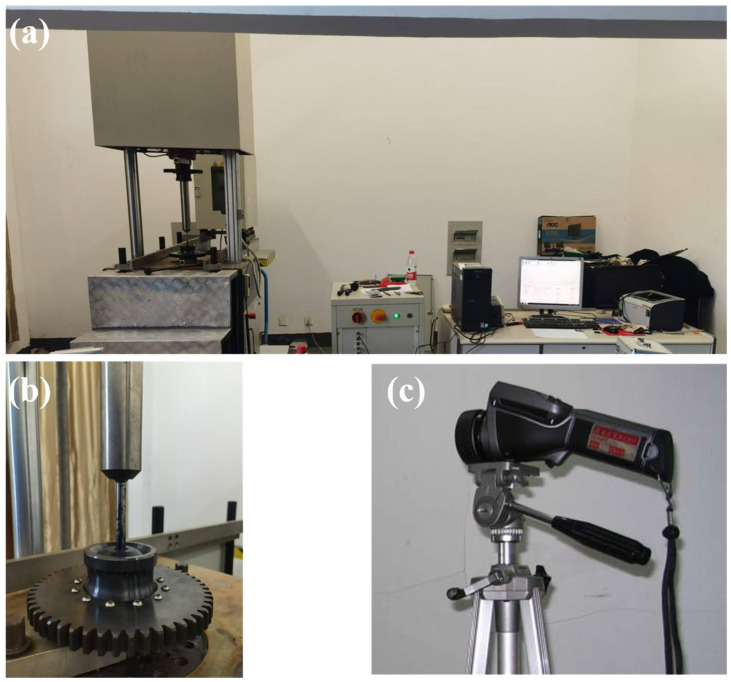
Experimental facilities adopted in this study. (**a**) High-frequency fatigue-testing machine and the controlling system, (**b**) specimen mounted in a gripping system, (**c**) the infrared camera (320 × 240 pixels (resolution), 0.05 °C at 30 °C (sensitivity/NETD), 60 Hz (image update rate)).

The traditional fatigue experiment was performed on a resonating testing machine (ZWICK-ROELL, Ulm, Germany) at room temperature of *T* = 24 °C, as shown in Figure 2a, with a loading frequency of about 80 Hz and the stress ratio *R* = −1. For typical fatigue-loading testing, a higher value of stress amplitude equal to 1000 MPa was first used, and then its value was reduced step-by-step until 800 MPa. Due to the large scatter of fatigue lifetimes at the high-cycle and very-high-cycle fatigue ranges, several specimens with similar stress amplitudes were added. To remove scratches from the surfaces of specimens, the surfaces of the specimens were polished with grade 600#, 1000#, 1500#, and 2000# abrasive papers, successively. The infrared thermography fatigue experiments were also implemented with this testing machine, and the infrared camera adopted was a Fluke Ti480Pro (Everett, WA, USA), as shown in Figure 2b. The main properties of the infrared camera were as follows: 320 × 240 pixels (resolution), 0.05 °C at 30 °C (sensitivity/NETD), 60 Hz (image update rate). In the infrared thermography fatigue experiments, step-by-step loading blocks with stress amplitudes of 500 MPa, 550 MPa, 600 MPa, 650 MPa, 700 MPa, 750 MPa, 800 MPa, 850 MPa, 900 MPa, 925 MPa, and 950 MPa were adopted. The loading period for each block was about 500 s, during which time the maximum temperature and temperature distribution along the horizontal axes of specimens were recorded by the infrared camera. To enhance the detectable capability of the specimens, black matt finish was coated uniformly on the horizontal parts of the specimens.

### 2.3. Theory of Fatigue Intrinsic Damping During Fatigue Loading

To obtain the intrinsic dissipation during fatigue loading, it is essential to establish the heat-convection equation of the specimens. According to heat-conduction theory, as long as the Biot number is smaller than 0.1, the temperature distribution along the radius direction can be taken as uniform. The Biot number (*B*i) is defined as follows:

(1) $$Bi=\frac{h_{c}l}{k}$$

where k=14.5 W/m·°C is the thermal conductivity of SAE52100. *l* is the characteristic dimension of the specimen, and it can be taken as the radius of the horizontal parts of the fatigue test specimen, *l* = 3 mm, in this study. *h*_c_ is the natural heat-convection coefficient, which is generally in the range of $5∼25\;W/m^{2}\cdot {}^{\circ}C$. Through the above calculations, it is seen that Bi≈0.015≪0.1. Thus, the temperature in each cross-section of the specimens can be taken a uniform one.

It is observed that the maximum value of the temperature increase is less than 10 °C in this study. Thus, the relative thermodynamic parameters such as elastic modulus (E), specific heat capacity (C), and heat conductivity (*k*) can be taken as constants during the equation deductions. Moreover, it is also believed that there is no coupling between the mechanical properties and temperature.

The loading frequency is relatively low, and the fatigue-damage evolution can be taken as a thermo-dynamic process under quasi-static conditions, in which the heat-convection equation can be written as follows [45]:

(2) $$\rho C\dot{T}-\nabla \cdot \left(k:\nabla T\right)=d_{1}+S_{the}+S_{ic}+Q_{e}$$

(3) $$\begin{array}{l} d_{1}=\left(\sigma -\rho \frac{\partial \Psi }{\partial \varepsilon }\right):\dot{\varepsilon }-\rho \frac{\partial \psi }{\partial \alpha }\cdot \dot{\alpha }\\ S_{the}=\rho T\frac{\partial^{2}\psi }{\partial T\partial \varepsilon }:\dot{\varepsilon }\\ S_{ic}=\rho T\frac{\partial^{2}\psi }{\partial T\partial \varepsilon }:\dot{\alpha } \end{array}$$

where *d*_1_ refers to the heat source caused by intrinsic dissipation, *S*_the_ refers to the heat source caused by the thermoelastic effect, *S*_ic_ refers to the heat source caused by coupling of the material’s mechanical parameter and temperature. It is recognized that the temperature variation caused by loading-stress amplitude is generally lower than 0.1 °C, which can be ignored during a full loading cycle. *S*_ic_ can also be taken as 0, for there is no change in material microstructures due to low temperature variation.

*Q*_e_ refers to the heat exchange between the specimens and the environment, which includes the heat conduction through the gripped parts of specimens and the heat convection to the environment. While the temperature variation is relatively low and not dramatic, it can be depicted with a simple linear relation, as follows:

(4) $$Q_{e}=-\rho C\frac{\theta }{\tau }$$

where $\theta =T-T_{0}$ refers to temperature variation, and τ refers to the heat-loss time constant. Thus, Equation (2) can be simplified as follows [45]:

(5) $$\rho C\left(\frac{\partial \theta }{\partial t}+\frac{\theta }{\tau }\right)-k\Delta \theta =d_{1}$$

During the temperature-stable stage, the temperature does not change with time, that is ∂θ∂t=0. Meantime, the temperature only changes along the horizontal direction, and it leads to

(6) $$\rho C\frac{\theta }{\tau }-k\frac{\partial^{2}\theta }{\partial x^{2}}=d_{1}$$

The solution of Equation (6) can be expressed as

(7) $$\theta \left(x\right)=A_{1}e^{rx}+A_{2}e^{-rx}+A3$$

where *A*_1_ and *A*_2_ are constants determined by boundary conditions and initial values, $r=\sqrt{\rho C/k\tau }$, and $A_{3}=\tau d_{1}/\rho C$. It is seen that the value of intrinsic dissipation can be derived as follows [45]:

(8) $$d_{1}=\frac{\rho C}{\tau }A_{3}=kr^{2}A_{3}$$

Equation (7) gives the distribution of temperature along the horizontal directions of specimens, from which *A*_1_, *A*_2_, *r*, and *A*_3_ can be identified by the experimental results. With these parameters, the intrinsic energy dissipation can be obtained according to Equation (8).

## 3. Results and Discussions

### 3.1. Traditional Fatigue-Testing Results

**Figure 3:**
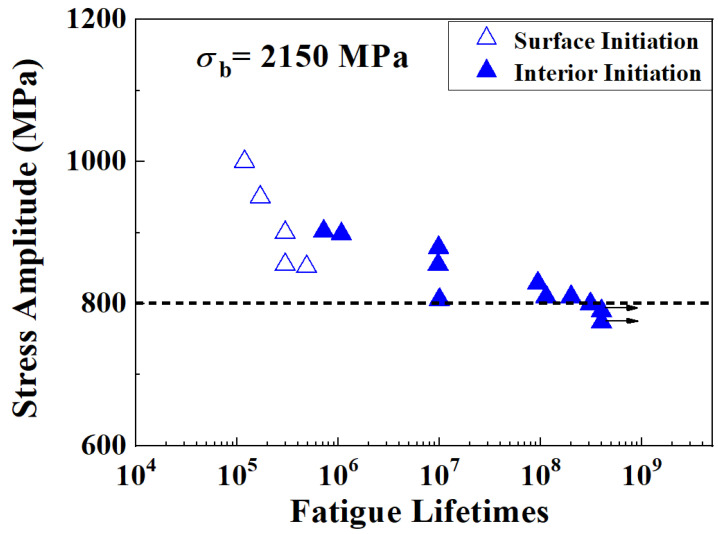
Fatigue data from the specimens under traditional frequency loading (UL). → indicates unbroken specimens.

The experimental data from traditional fatigue testing are shown in Figure 3, where the hollow triangle symbols, “△”, represent the surface-initiation failure mode, while the solid triangle symbols, “▲”, represent the interior-initiation failure mode. There are also two unbroken specimens loaded up to a fatigue lifetime of *N* = 4 × 10^8^, which lasted for 58 days and are labeled with an arrow, “→”. When the applied stress amplitudes are large, the cracks initiate from the specimen surface and lead to relatively shorter fatigue lifetimes, while at smaller stress amplitudes, most of the specimens break from the interior-initiation defects and the fatigue lifetimes are much longer. If the stress amplitude corresponding to a fatigue lifetime of *N* = 1 × 10^7^ is taken as the fatigue limit, the fatigue limit is about 800 MPa.

**Figure 4:**
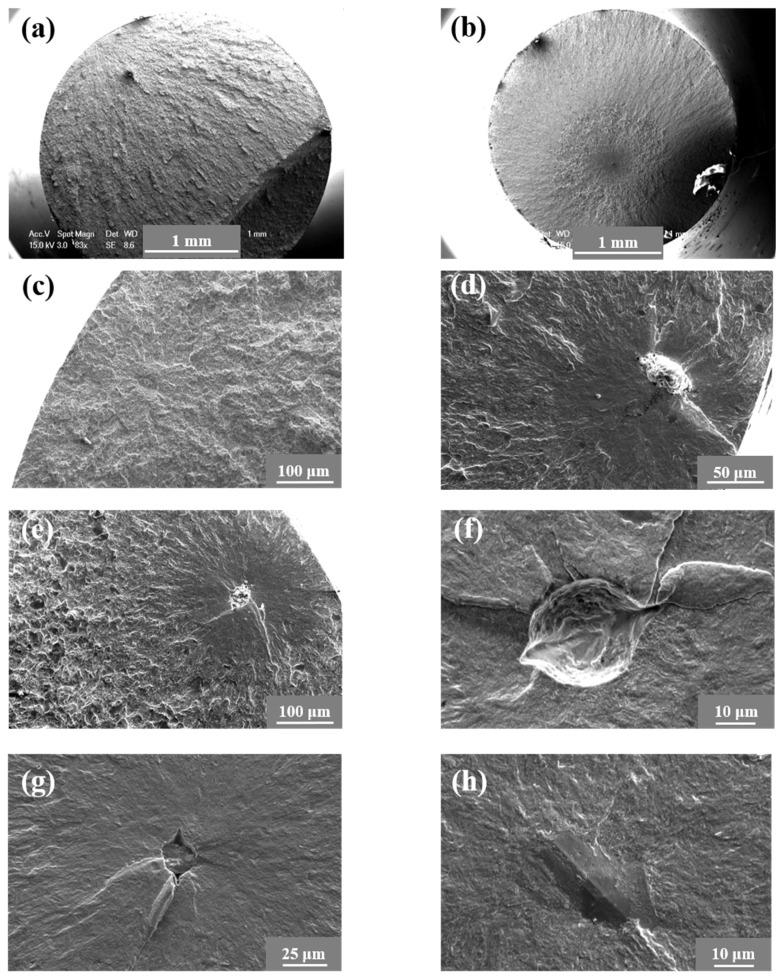
Typical morphologies of fractographies for different specimens. (**a**) General view of surface-crack initiation; (**b**) general view of interior-crack initiation; (**c**) σ = 950 MPa, *N_f_* = 1.688 × 10^5^; (**d**) σ = 902 MPa, *N_f_* = 7.168 × 10^5^; (**e**) σ = 898 MPa, *N_f_* = 1.072 × 10^6^; (**f**) σ = 829 MPa, *N_f_* = 9.387 × 10^7^; (**g**) σ = 810 MPa, *N_f_* = 1.131 × 10^8^; (**h**) σ = 800 MPa, *N_f_* = 3.103 × 10^8^.

The typical fractographies of broken specimens are illustrated in Figure 4. At a stress amplitude of 950 MPa, the specimens broke quickly and presented surface-failure mode, as shown in Figure 4a. At relatively lower stress amplitudes, such as 902 MPa and 898 MPa, the specimens presented subsurface-crack-initiation failure modes, as shown in Figure 4b,c, where clear evidence of inclusions is displayed. Interior-crack-initiation failure modes were mostly observed as presented in Figure 4d–f, where both big inclusions and grains would act as crack-initiation sources.

### 3.2. Temperature Variation of Specimens and Determination of Fatigue Limits

**Figure 5:**
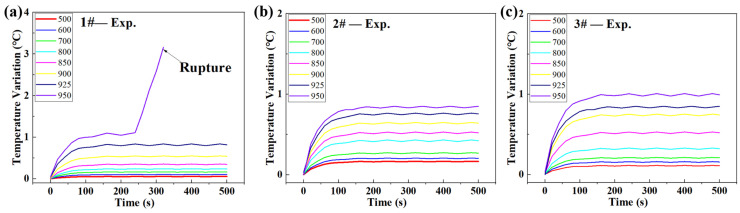
The temperature-variation evolutions for three specimens loaded at different stress-amplitude blocks. (**a**) Specimen 1#, (**b**) Specimen 2#, (**c**) Specimen 3#.

**Figure 6:**
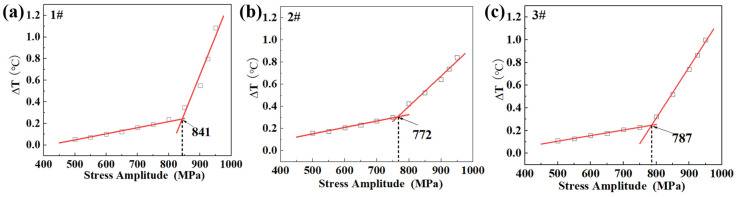
The variations of the maximum temperature increase at different values of stress amplitude and the determination of fatigue limits. (**a**) Specimen 1#, (**b**) Specimen 2#, (**c**) Specimen 3#.

The temperature-variation evolution during fatigue loading at each loading block is displayed in Figure 5. The recorded time was about 500 s, during which time the temperature rise stabilized. Three specimens were tested, and they all presented the same tendency. The temperatures of specimens would increase abruptly, then achieve a stable stage. The times needed for stabilization were different for each loading-stress amplitude, but it was seen that the longest time was no longer than 200 s, corresponding to fatigue-loading cycles of 16,000 cycles. Although much longer fatigue-loading times were also feasible, it should be noted that longer loading times would lead to large fatigue-damage accumulation at large fatigue-loading-stress amplitudes, which was not desired in detecting the fatigue limits of the specimens. Thus, a recording time of 500 s was adopted in this study. Besides, the stabilized temperature increased with the stress amplitude, and the maximum temperature variation at each stress amplitude was recorded and is presented in Figure 6. The first specimen presented a slightly higher temperature rise than those of the other two specimens, and it was fractured during the highest loading block.

As illustrated in Figure 6, the temperature variation can be divided into two linear parts: the temperature variation increased slowly at first, and then, at a turning point, it increased abruptly. Through linear fitting of the two groups of data, the conjunction point was considered the fatigue limit. The fatigue limits given by the three specimens are 841 MPa, 772 MPa, and 787 MPa, respectively. Unlike the first specimen, the second and third specimens present similar prediction results, and they are much closer to that of traditional fatigue-testing results, 800 MPa. After careful examination of the first temperature-variation curve, it was noticed that the temperature variations at stress amplitudes of 750 MPa, 800 MPa, and 850 MPa were not so distinguishable as those of the other two specimens. Thus, the prediction results would bring large deviations. If the fatigue limit obtained from traditional testing results was taken as 800 MPa, the errors in predictions from infrared thermography were 41 MPa, 28 MPa, and 13 MPa. It was concluded that the infrared thermography presented good predictions.

### 3.3. The Derivation of Intrinsic Dissipation Based on Temperature Distribution

**Figure 7:**
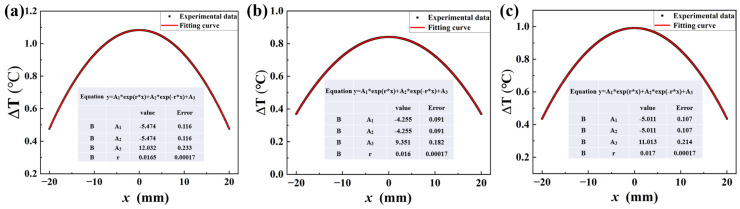
The temperature distribution of specimens along the horizontal directions and data fitting according to Equation (7). (**a**) Specimen 1#, (**b**) Specimen 2#, (**c**) Specimen 3#.

It can be seen from Equation (8) that the temperature distribution is essential for the calculation of fatigue intrinsic dissipation. Thus, the temperature distribution for each loading case was recorded, and the typical temperature distributions for three specimens are presented in Figure 7. They were all fitted according to Equation (7), and the corresponding parameters are also listed in the figures. It was noticed that *r* and *A*_3_ were the two parameters determining the value of intrinsic dissipation, *d*_1_. These two values were different for different specimens. Moreover, it should be noticed that the fitting parameters might be different, even for the same specimen at different loading cases.

**Figure 8:**
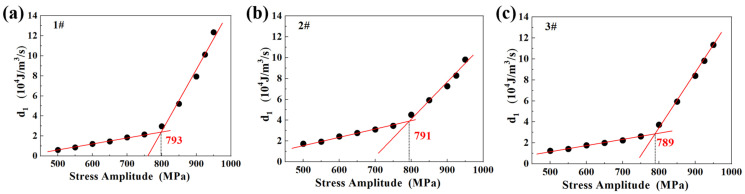
The calculated intrinsic damping at different stress amplitudes and determination of fatigue limits. (**a**) Specimen 1#, (**b**) Specimen 2#, (**c**) Specimen 3#.

The values of intrinsic dissipation for each specimen are presented in Figure 8. It was also revealed that values of intrinsic dissipation could be divided into two groups. Different from the superficial explanation of temperature variation, intrinsic dissipation could be related to the failure mechanism directly. As suggested in some previous studies [38,43,45], the intrinsic dissipation at low stress amplitudes generally corresponds to microstructural degradation limited to some local grains, which would not lead to the formation of macro plastic deformation, and the values are generally small. Beyond a certain stress amplitude, the microstructural degradation induced by cyclic loading would pass through the limits of grain boundaries and result in overall microstructural degradation, which resulted in final failure. Beyond the stress amplitude, the mechanism of intrinsic dissipation changed and increased abruptly. Through linear data fitting, the conjunction point could also be taken as the fatigue limit. As shown in Figure 8, the fatigue limits determined by intrinsic dissipation were 793 MPa, 791 MPa, and 789 MPa, respectively. It was obvious that the predictions obtained through intrinsic dissipation were much closer to those of traditional fatigue-testing results than those obtained through maximum temperature variation, suggesting that intrinsic dissipation could serve as a much more reasonable parameter for the prediction of fatigue limits.

### 3.4. The Simulation of Self-Heating in Fatigue Loading

**Figure 9:**
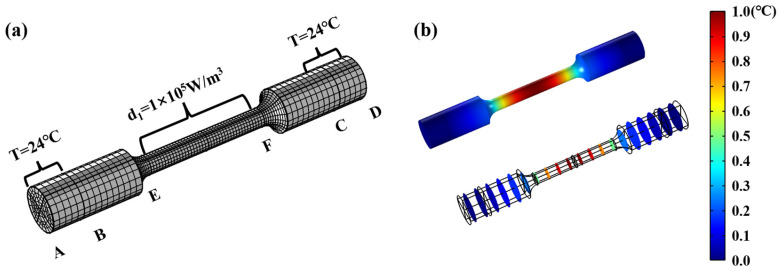
The FEM simulation of self-heating in fatigue loading. (**a**) The boundary setting and mesh grid of the specimen. (**b**) The temperature-field map.

In the introduction of the theoretical background for self-heating in fatigue loading, several assumptions were adopted. Due to the limits of experimental setups, these assumptions were hard to justify. Thus, it was necessary to examine the rationalities of these assumptions. FEM simulation provided an appropriate avenue to fulfill and verify these assumptions. Adopting the great functionality of multiphysics coupling, COMSOL Multiphysics was adopted for self-heating during fatigue loading. Illustrations of the setups are presented in Figure 9a. Hexahedron meshes were adopted, and the total number is 7650. The main coefficients adopted were the surface-heat radiation ratio, *ε* = 0.95, the heat-conduction coefficient of specimens with environment *hc* = 5 W/(m^2^·K), and the thermal conductivity of SAE52100, *h* = 5 W/(m^2^·K). The sections of AB and CD were taken as gripping parts, where they were set as fixed, and the temperature was set at 24 °C. Intrinsic dissipation was taken as the heat source for the temperature rise of specimens, and it was set on the horizontal parts of the specimens. Different stress amplitudes corresponded to different intrinsic dissipations, and the values are shown in Figure 8. Taking specimen 3# as an example, the temperature-field map is presented in Figure 9b. It was seen that the maximum temperature rise occurred on the middle points of the specimens. The temperature along the cross-section is also presented in Figure 9b, indicating that the temperature was uniform in the radial directions of the specimens. The simulation results agreed well with the assumption of the Bi number.

**Figure 10:**
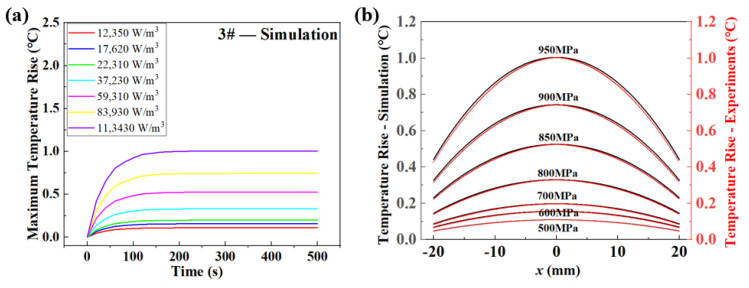
A comparison between the predicted fatigue life and the experimental results, showing the simulation results for temperature-variation evolution and temperature distribution at different input heat sources or loading-stress amplitudes. (**a**) Numerical simulations of maximum temperature rising with time at different input intrinsic dissipations. (**b**) The numerical simulations of temperature distributions along the horizontal directions of specimens at different applied load.

The evolution of temperature variation is also illustrated in Figure 10a. The simulation results indicated that the temperature increased quickly in the first loading of 100 s and then stabilized. Higher-input intrinsic dissipation or stress amplitude led to a higher stabilized temperature. It was obvious that the simulations presented almost the same results as those of the experiments, suggesting that the simulations could reflect the temperature-evolution process of fatigue loading. Besides, the temperature distributions along the horizontal directions of specimens were also extracted from the simulations, as shown in Figure 10b. Seven temperature distributions were plotted and compared with those of the experimental results, which presented great coincidence. Supported by the simulation results, the assumptions and validities of the theoretical model were verified.

## 4. Conclusions

Infrared thermography techniques have proven to be very effective for assessing the fatigue limits of metallic materials with obvious temperature variations. But for some materials, it has been shown that the temperature variation is very limited, and the accuracy of infrared thermographic techniques is not verified. In this study, the fatigue properties of a high-strength steel (SAE52100) were evaluated with traditional fatigue-loading techniques and infrared thermographic methods. FEM simulation of temperature variation during fatigue loading was implemented to verify experimental results. The main results are summarized as follows.

(1) The typical fatigue experiments were carried out at a frequency of 80 Hz, and the fatigue limit of the material was about 800 MPa. Surface-initiation failure modes appeared at higher stress amplitudes and lower fatigue lifetimes, while interior failure modes initiating from inclusions or big grains occurred at lower stress amplitudes and higher fatigue lifetimes.

(2) Three additional specimens were loaded with step-by-step increasing stress-loading amplitudes, where the maximum temperature increments and temperature distributions were recorded via infrared thermographic techniques. The infrared detections revealed that the maximum temperature rise was only about 1 °C. The fatigue limit was first evaluated based on the maximum temperature variation. The fatigue limits predicted with the maximum temperature variation were shown to be 841 MPa, 772 MPa, and 787 MPa, respectively.

(3) Based on the temperature distribution curves of the specimens, the values of intrinsic dissipation were obtained and adopted for the prediction of fatigue limits. The fatigue limits predicted based on fatigue intrinsic dissipation were 793 MPa, 791 MPa, and 789 MPa, respectively. The predictions obtained through intrinsic dissipation were much closer to those of traditional fatigue-testing results than those obtained through the maximum temperature variation, suggesting that intrinsic dissipation could serve as a much more reasonable parameter for the prediction of fatigue limits.

(4) Finally, an FEM simulation of temperature variation during fatigue loading was implemented to verify the experimental results and theoretical models. Supported by the simulation results, the assumptions and validities of the theoretical model were verified. This study provides a solid foundation for the applications of infrared thermography technique for materials with lower energy dissipations.

The infrared thermographic method presents an accurate and quick method for determining the fatigue limits of new materials, which is verified by the studies. But, it should be pointed out the fatigue data cannot be obtained from the results, which is not applicable for occasions where the fatigue strength at specific fatigue lifetimes is needed.

## Figures and Tables

**Table 1:** Chemical composition of the tested SAE52100 steel (wt.%).

C	Cr	Mn	Si	P	S	Fe
1.01	1.45	0.35	0.28	0.015	0.01	Balance

**Table 2:** Mechanical properties of the tested SAE52100 steel (wt.%).

Micro-Hardness Hv (kgf/mm^2^)	Young’s Modulus (GPa)	Yield Strength (MPa)	Tensile Strength (MPa)
801	207.2	2032	2272

## Data Availability

The data presented in this study are available on request from the corresponding author. The data are not publicly available due to requirements of the Corporation.
